# MicroRNA-874-3p Aggravates Doxorubicin-Induced Renal Podocyte Injury via Targeting Methionine Sulfoxide Reductase B3

**DOI:** 10.1155/2020/9481841

**Published:** 2020-08-18

**Authors:** Yan Dai, Meng Gao, Linxia Li, Zhang Mao, Lina Xu, Lianhong Yin, Yan Qi, Jinyong Peng

**Affiliations:** ^1^College of Pharmacy, Dalian Medical University, No. 9 West Section Lvshun South Road, Dalian, China; ^2^Key Laboratory for Basic and Applied Research on Pharmacodynamic Substances of Traditional Chinese Medicine of Liaoning Province, Dalian Medical University, Dalian, China; ^3^Institute (College) of Integrative Medicine, Dalian Medical University, No. 9 West Section Lvshun South Road, Dalian, China; ^4^National-Local Joint Engineering Research Center for Drug Development (R&D) of Neurodegenerative Diseases, Dalian Medical University, Dalian, China

## Abstract

Clinical application of doxorubicin (Dox) is limited due to its serious side effects including nephrotoxicity, and kidney podocytes play important roles in renal diseases. MicroRNAs (miRNAs) are critical regulators associated with human diseases. The purpose of this study was to explore a novel target in adjusting Dox-induced renal podocyte injury. Through a double luciferase reporter gene experiment, it was found that miR-874-3p directly targeted methionine sulfoxide reductase B3 (MsrB3). During the tests of miR-874-3p inhibitor and MsrB3 siRNA in human podocytes or miR-874-3p antagomir in mice, we found that the expression levels of downstream oxidative stress and apoptosis-related proteins were regulated by miR-874-3p/MsrB3 signal to alleviate or aggravate renal podocyte injury. The data in the present work showed that miR-874-3p aggravated Dox-caused renal podocyte injury by promoting apoptosis and oxidative damage via inhibiting MsrB3. Therefore, miR-874-3p/MsrB3 should be considered as a new therapeutic target in controlling renal podocyte injury induced by Dox.

## 1. Introduction

Doxorubicin (Dox), an antitumor anthracycline antibiotic, has been widely used to treat various types of cancers including prostate cancer, hematological malignancies, bile duct neoplasms, stomach cancer, uterus cancer, and liver cancer [1–3]. However, the clinical application of Dox is limited owing to its adverse reactions in which nephrotoxicity is one serious side effect produced by it [4–6]. However, the mechanisms of Dox-induced nephrotoxicity are still not entirely clearly understood. Therefore, investigating molecular mechanisms and exploring effective drug targets in controlling Dox-induced renal injury are of great significance.

Podocytes are highly differentiated epithelial cells on the outer surface of glomerular capillaries [7], which play crucial roles in glomerular diseases. Podocyte injury has been considered as one critical factor in glomerular filtration barrier dysfunction, and continuous podocyte injury can lead to proteinuria, glomerulosclerosis, and renal impairment [8–10]. In addition, glomerulosclerosis, the main pathological process, can cause end-stage renal disease, and podocyte injury is crucial for the progression of glomerular disorders [11]. Therefore, exploring novel therapeutic targets to prevent and treat kidney injury via targeting podocyte is urgent.

Previous studies have shown that the biological processes of oxidative stress, apoptosis, and inflammation are involved in Dox-induced renal podocyte injury. Some reports have shown that C-X-C chemokine receptor type 4 plays important roles in mediating oxidative stress-caused podocyte injury [12]. Sirtuin (Sirt) 6 can alleviate podocyte injury via anti-inflammatory and antiapoptotic actions [13]. Microsomal PGE synthase- (mPGES-) 1 can aggravate adriamycin- (ADR-) induced podocyte injury via triggering inflammatory response [14]. It has been confirmed that oxidative stress-induced podocyte injury can cause the development of kidney diseases [15]. Moreover, increasing studies have indicated that the apoptosis of podocyte shows major roles in the pathogenesis of proteinuria and the progression of chronic nephropathy [16]. Hence, regulating oxidative stress and apoptosis should be the effective method to treat Dox-induced podocyte injury.

MicroRNAs (miRNAs) can suppress gene expression through binding with the 3′-untranslated regions (UTRs) of their targeted mRNAs [17, 18]. Previous works have reported that inhibition of miR-155 can alleviate podocyte injury [19]. miR-27a can aggravate podocyte injury via adjusting peroxisome proliferator-activated receptor *γ*- (PPAR*γ*-) mediated *β*-catenin activation in diabetic nephropathy [20]. miR-377 can aggravate podocyte injury through adjusting oxidative stress and inflammation [21]. Therefore, exploring novel miRNAs as the potent drug targets may be the important ways to regulate Dox-induced renal podocyte injury.

miR-874-3p is the mature product of miR-874 [22]. A growing number of studies have shown that miR-874 has important parts in diseases via targeting different genes. Inhibition of miR-874 can protect heart ischemia-reperfusion injury via targeting transcription 3 (STAT3) [23]. miR-874-3p shows a tumor-suppressive role by promoting apoptosis in hepatocellular carcinoma (HCC) via targeting peptidylprolyl cis-trans isomerase NIMA-interacting1 (PIN1) [24]. miR-874 can suppress tumor proliferation in hepatocellular carcinoma by targeting the *δ* opioid receptor (DOR) [25]. However, the effect of miR-874-3p on Dox-induced renal podocyte injury has not been reported.

It has been reported that there are three methionine sulfoxide reductase B (MsrB) genes including MsrB1, MsrB2, and MsrB3 in human and mouse genomes [26]. MsrB1 in the cytosol and nucleus has the highest enzyme activity owing to the presence of selenocysteine in its active site. MsrB2 in mitochondria has a strong affinity to methionine-R-sulfoxide (Met-R-SO) [27]. MsrB3 gene has two forms including MsrB3A and MsrB3B [26]. Some researches have shown that MsrB3 has antioxidant and antiapoptotic effects. The depletion of MsrB3 can induce cancer cell apoptosis via adjusting the production of reactive oxygen species (ROS) and the activation of the intrinsic mitochondrial pathway [28]. MsrB3 deficiency can induce cancer cell apoptosis through ER stress-dependent and p53-independent pathways [26], which can also inhibit cell growth via activating p53-p21 and p27 pathways [29]. However, the function of MsrB3 on Dox-induced renal podocyte injury has not been reported, and the relationship between miR-874-3p and MsrB3 has not been reported yet.

Thus, the purpose of this study was to explore the molecular mechanisms of miR-874-3p on Dox-induced renal podocyte injury via targeting MsrB3 and then to explore novel targets for new drug research and development.

## 2. Materials and Methods

### 2.1. Chemicals and Materials

Doxorubicin (Dox) was manufactured by Sigma (Santa Clara, CA, USA). 4′,6′-Diamidino-2-phenylindole (DAPI) was produced by Sigma (St. Louis, MO, USA). 3-(4,5-Dimethylthiazol-2-yl)-2,5-diphenyl tetrazolium bromide (MTT) was purchased from Roche Diagnostics (Basel, Switzerland). Proteinuria, creatinine (Cr), urea nitrogen (BUN), glutathione (GSH), glutathione peroxidase (GSH-Px), MDA detection, and superoxide dismutase (SOD) kits were manufactured by Nanjing Jiancheng Institute of Biotechnology (Nanjing, China). ROS assay kit, phenylmethanesulfonyl fluoride (PMSF), cell lysis buffer, and bicinchoninic acid (BCA) protein assay kit were all provided by Beyotime Biotechnology (Jiangsu, China). MicroRNAs Quantitation PCR Kit, MicroRNA First Strand cDNA Synthesis Kit, and SanPrep Column MicroRNA Mini-Preps Kit were provided by Sangon Biological Engineering Technology & Services Co., Ltd. (Shanghai, China). Double-Luciferase Reporter Assay Kit was provided by Promega Biotech (Beijing, China). Tissue Protein Extraction Kit was obtained from KEYGEN Biotech. Co., Ltd. (Nanjing, China). The terminal deoxynucleotidyl transferase dUTP nick-end labeling (TUNEL) assay kit, TransZolTM, TransScript® All-in-One First-Strand cDNA Synthesis SuperMix for qPCR (One-Step gDNA Removal), and TransStart® Top Green qPCR SuperMix were obtained from Beijing TransGen Biotech Co., Ltd. (Beijing, China). Lipofectamine 2000 was obtained from Thermo Fisher Scientific (Shanghai, China). Wild-type MsrB3 (MsrB3-WT) and 3′-UTR-mutated MsrB3 (MsrB3-MUT) luciferase reporter plasmids, miR-874-3p mimic, miR-874-3p inhibitor, miR-874-3p antagomir, and their negative control (NC) oligos were all purchased from RiboBio Co., Ltd. (Guangdong, China). MsrB3 siRNA was obtained from General Biosystems (Anhui, China). MsrB3 overexpression plasmids were purchased from GeneCopoeia, Inc. (USA).

### 2.2. Extraction of Mouse Primary Podocytes and Cell Culture

Primary renal podocytes were obtained from young male C57BL/6J mice, which were purchased from the Experimental Animal Center at Dalian Medical University (Dalian, China) (SCKK: 2018-0003). Briefly, the mice were executed by cervical dislocation, and both kidneys were removed under sterile conditions and placed in a precooled phosphate-buffered solution (PBS). Kidney medulla was removed, and only cortex was collected. Glomeruli were separated by differential screening. The KI-3T3 medium (DMEM/F12 medium) and DMEM medium (high sugar type) were mixed at the volume ratio of 1 : 1 with 100 *μ*g/mL of streptomycin and 100 unit/mL of penicillin, and 10% (*v*/*v*) fetal bovine serum (FBS) was used for suspending the glomeruli, which were then grown in culture flasks in an atmosphere of 5% CO_2_ at 37°C [30, 31]. Human podocytes were provided by Kelei Biological Technology Co., Ltd. (Shanghai, China), and the cell lines were maintained in McCoy's 5A medium (Gibco, California, USA) supplemented with 100 unit/mL of penicillin, 100 mg/mL of streptomycin, and 10% FBS under 5% CO_2_, 21% O_2_ at 37°C. Immunofluorescence assay was used to prove that the cells extracted from mice were primary podocytes (Supplementary Figure S1).

### 2.3. Dox-Induced Cell Injury

Human podocytes and mouse primary podocytes were separately cultured in 96-well plates at a density of 5‐10 × 10^4^ cells/mL per well for about 24 h. Human podocytes were administered with different concentrations of Dox (0, 8, 16, 32, and 48 *μ*M) for different times (12, 24, and 48 h), and mouse primary podocytes were administered with different concentrations of Dox (5, 10, 20, 30, 50, and 100 *μ*M) for 3, 12, and 24 h. Afterwards, 10 *μ*L of MTT (5 mg/mL) solution was added to each well in the dark conditions. After incubation at 37°C for 4 h, MTT solution was removed and 150 *μ*L of dimethyl sulphoxide (DMSO) was added to each well. The absorbance was determined at 490 nm by a POLARstar OPTIMA multidetector microplate reader from Bio-Rad, San Diego, USA.

### 2.4. Measurement of Intracellular ROS

Human podocytes and mouse primary podocytes were inoculated into 24-well culture plates at a density of 5‐10 × 10^4^ cells/mL. On the next day, human podocytes were challenged with 32 *μ*M of Dox for 24 h, and mouse primary podocytes were challenged with 20 *μ*M of Dox for 12 h. After that, the medium was removed, and 500 *μ*L of serum-free medium (10 *μ*M of DCFH-DA) was added to each well. After incubation at 37°C for 25 min, the images were captured by one inverted fluorescence microscope (Olympus, Tokyo, Japan).

### 2.5. Animals and Dox-Induced Kidney Injury *In Vivo*

Male BALB/c aged 6-8 weeks and weighing 18-22 g were purchased from the Experimental Animal Center of Dalian Medical University (Dalian, China) (SCKK: 2018-0003). All experimental procedures were carried out in accordance with the People's Republic of China Legislation Regarding the Use and Care of Laboratory Animals and approved by the Animal Care and Use Committee of Dalian Medical University. Animals were housed in a room with controlled temperature (25 ± 2°C), and there are 2-3 mice in each cage for 12 h of a light/dark cycle and free access to water and food. Before the experiment, they were allowed one week to acclimate to the environment. Sixteen mice were randomly divided into two groups (*n* = 8): control group and Dox group. The model group was induced by Dox at the dose of 10 mg/kg via single tail-vein injection [32, 33], while the control mice were received equivalent 0.9% saline. After two weeks, all mice were sacrificed, and blood and kidney samples were collected. The blood samples were centrifuged (3500 r/min, 4°C) for 10 min, and the supernatant was collected and placed at -80°C. Parts of kidney tissues were immobilized in 4% paraformaldehyde, and the rest of kidney tissues were stored at −80°C for subsequent experiments.

### 2.6. Measurement of Cr, BUN, SOD, MDA, GSH, and GSH-Px Levels

The urea levels of proteinuria in mice during 24 h treatment, the serum Cr and BUN levels, and the levels of SOD, MDA, GSH, and GSH-Px in kidney tissues of mice were detected using the appropriate kits according to the instructions.

### 2.7. Histopathologic Assay

The kidney tissue was fixed in 4% paraformaldehyde, made into paraffin sections about 5 *μ*m thick, and stained with hematoxylin-eosin (H&E). Images of the stained sections were obtained using one optical microscope (Nikon Eclipse TE2000-U, Japan) with 200x magnification.

### 2.8. TdT-Mediated dUTP Nick-End Labeling (TUNEL) Assay

According to the instructions, apoptosis detection was executed using the TUNEL assay kit. *In vivo*, the paraffin tissue sections were dewaxed in xylene liquid, washed with gradient concentrations of ethanol and PBS, permeated with 0.5% Triton-100, and incubated with 52 *μ*L of reaction mixture (2 *μ*L TdT+50 *μ*L labeling solution) under 37°C for 1 h. Finally, the kidney tissue sections were rinsed with PBS three times, permeated with 0.5% Triton-100, and sealed with a coverslip. *In vitro*, the cells were exposed to Dox, rinsed with PBS, fixed in 4% paraformaldehyde for 20 min, and permeated with 0.5% Triton-100 in 24-well plates. Then, the TUNEL mixture (200 *μ*L) was added into each well under 37°C of humid and light avoidance conditions for 1 h. Finally, the images were captured by fluorescent microscopy (Olympus, Japan) with 200x magnification.

### 2.9. Detection of miR-874-3p Levels *In Vivo* and *In Vitro*

Total miRNA from human podocytes, mouse primary podocytes, and kidney tissues of mice were extracted using the SanPrep Column MicroRNA Mini-Preps Kit. The purity of the miRNA mentioned above was determined using a micronucleic acid protein analyzer (Biochrom, USA). The RT-PCR was performed using a MicroRNA First Strand cDNA Synthesis Kit based on the manufacturer's protocol. The expression levels of miR-874-3p were detected by a CFX96 PCR system (Bio-Rad Laboratories, USA) with SYBR Green Master Mix using a MicroRNAs Quantitation PCR Kit based on the manufacturer's protocol. The primers of miRNA are listed in Supplemental Table S1. U6 (Sangon Biological Engineering Technology & Services Co., Ltd., China) is universal for miRNA.

### 2.10. Dual-Luciferase Reporter Assay

The plasmids which contain the putative (wild-type, MsrB3-WT) or mutated binding site for miR-874-3p and MsrB3 were synthesized. Then, they were cloned into a pmiR-report vector. The miR-874-3p binding site was mutated from 5′-CAGGGCAA-3′ to 5′-GTCCCGTT-3′. For luciferase assay, human podocytes were inoculated into 24-well plates, and on the next day, miR-874-3p mimic or miR-874-3p mimic negative control and plasmid DNA (wt-Luc-MsrB3, mut-Luc-MsrB3) were cotransfected into the cells. After 24 h of transfection, the cells were collected and the luciferase activity was estimated by using the Dual-Light Chemiluminescent Reporter Gene Assay System (Berthold, Germany), which was normalized to firefly luciferase activity following the instructions of the kit.

### 2.11. Cotransfection Test of MsrB3 and miR-874-3p

Human podocytes were inoculated into 6-well plates for 24 h at a density of 5‐10 × 10^4^ cells/mL. MsrB3 overexpression plasmid negative control (NC) and miR-874-3p mimic negative control (NC) and MsrB3 overexpression plasmid and miR-874-3p mimic were cotransfected into the cells. MsrB3 overexpression plasmid was also transfected. After transfection for 24 h, the mRNA and protein levels of MsrB3 were detected.

### 2.12. Quantitative RT-PCR Assay

Total RNA samples were extracted from human podocytes, mouse primary podocytes, and kidney tissues of mice by TranZol. The purity of the extracted RNA samples was detected using a micronucleic acid protein analyzer (Biochrom, USA). According to the manufacturer's protocol, each RNA sample was reverse transcribed into cDNA using a TransStart Top Green qPCR SuperMix kit. RT-PCR was performed with SYBR Green Master Mix used the CFX96 PCR system (Bio-Rad Laboratories, USA). The forward primers (F) and reverse (R) primers of RNA are listed in Supplementary Table S2. GAPDH is used to normalize mRNA levels [34].

### 2.13. Western Blotting Assay

Protein extraction kit was used to extract total protein from human podocytes, mouse propodocytes, and mouse kidney tissues. According to the manufacturer's protocol, the concentration of the extracted protein was measured by a BCA protein detection kit. Then, the protein samples were loaded into SDS-PAGE (8%-12%), transferred to PVDF membranes (0.45 *μ*m, Millipore, USA), blocked with 5% defatted milk or 3% BSA-PBS for 2 h, and incubated with diluted primary antibodies at 4°C. On the next day, the primary antibodies were removed, and the PVDF membranes were incubated with the diluted secondary antibody at room temperature for 2 h. Finally, the bands of the proteins were detected by ChemiDoc XRS (Bio-Rad, USA) and their expression levels were assayed by the enhanced chemiluminescence method. The primary antibodies are given in Supplemental Table S3. GAPDH was used to normalize the relative protein levels [34].

### 2.14. Inhibitor Transfection of miR-874-3p *In Vitro*

In order to verify the role of miR-874-3p on Dox-induced podocyte injury, the experiment of miR-874-3p inhibitor transfection was designed *in vitro*. Briefly, Lipofectamine 2000 was diluted with serum-free medium and equilibrated at room temperature for 5 min. In addition, miR-874-3p inhibitor negative control and miR-874-3p inhibitor were separately diluted with serum-free medium. Then, the diluted Lipofectamine 2000 was mixed with diluted miR-874-3p inhibitor and its negative control gently and balanced for 20 min at room temperature to form liposomes. Finally, the transfection mixture was added into the human podocytes and mouse primary podocytes. After incubation at 37°C for 5 h, the cell medium was replaced with FBS. After transfection for 24 h, the projects including ROS level, cell apoptosis, the expression level of miR-874-3p, the mRNA level of MsrB3, and the protein levels of MsrB3, SOD2, NQO1, Bax, and Bcl-2 were detected.

### 2.15. Antagomir Transfection of miR-874-3p *In Vivo*

In order to further explore the function of miR-874-3p on Dox-induced renal podocyte injury, the experiment of miR-874-3p silencing *in vivo* was carried out. The methods were as follows: twenty male BALB/c mice were randomly divided into the control group, NC group, Dox group, and Dox+miR-874-3p antagomir group (*n* = 5). The mice in the Dox+miR-874-3p antagomir group and the NC group were injected with miR-874-3p antagomir (50 nmol) or the same dosage of miR-874-3p antagomir NC for 3 times (on day 7, day 10, and day 13) via the tail vein. The mice in the Dox group and the Dox+miR-874-3p antagomir group were injected with Dox (10 mg/kg) by the tail vein, whereas the mice in the control group and the NC group were injected with equivalent 0.9% saline. On day 14, all of them were sacrificed. The items including histopathological examination, the levels of Cr, BUN, SOD, MDA, GSH, GSH-Px, and cell apoptosis, the expression level of miR-874-3p, the mRNA level of MsrB3, and the protein levels of MsrB3, WT-1, nephrin, desmin, NQO1, SOD2, Bcl-2, and Bax were detected.

### 2.16. Transfection of MsrB3 siRNA *In Vitro*

MsrB3 siRNA transfection experiment was performed on human podocytes. The final concentrations of MsrB3 siRNA and MsrB3 siRNA negative control were 50 nM. The solutions were blended with Lipofectamine 2000 following the protocol to get the mixed reagents, which were balanced at room temperature for 20 min. Then, the human podocytes were transfected with mixed reagents, and after 24 h, cell apoptosis, ROS level, miR-874-3p expression level, MsrB3 mRNA level, and the protein levels of MsrB3, SOD2, NQO1, Bax, and Bcl-2 were detected.

### 2.17. Data Analysis

The data were presented as the mean ± SD. The statistical analysis was carried out by GraphPad Prism 6.01 software (GraphPad Software, Inc, CA, USA). When comparing two groups, the statistical analysis was executed with unpaired Student's *t*-test. When comparing multiple groups, the statistical analysis was performed with one-way analysis of variance (ANOVA) followed by Tukey's post hoc test, and *p* < 0.05 or *p* < 0.01 was considered to be statistical significance. The data and statistical analysis are in line with recommendations for pharmacological experimental design [35].

## 3. Results

### 3.1. Dox Causes Podocyte Injury *In Vitro* and *In Vivo*

**Figure 1:**
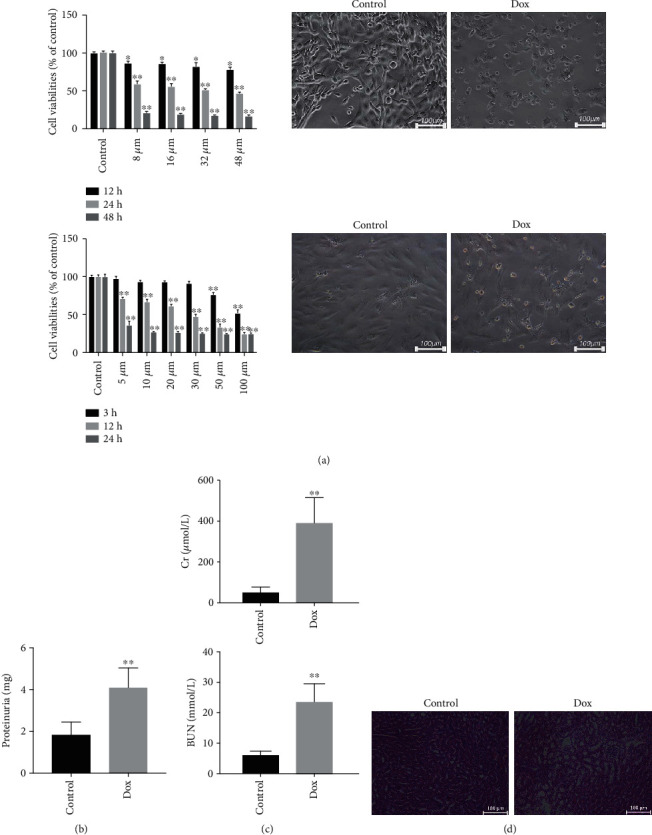
Dox causes podocyte injury *in vitro* and *in vivo*. (a) Cellular viability and morphology of human podocytes and mouse primary podocytes by MTT assay (*n* = 6). (b) The 24-hour proteinuria levels in mice caused by Dox (*n* = 8). (c) Serum levels of Cr and BUN in mice caused by Dox (*n* = 8). (d) H&E staining (×200 magnification) of the kidney in mice. All data are listed as the mean ± SD. ^∗^*p* < 0.05 and ^∗∗^*p* < 0.01 compared with control groups.

The data in Figure 1(a) showed that the cell viabilities of human podocytes exposed to Dox (32 *μ*M) for 24 h and mouse primary podocytes challenged with Dox (20 *μ*M) for 12 h were significantly reduced, and the morphology was obviously changed compared with control groups. As shown in Figure 1(b), the 24-hour urine level of proteinuria in the Dox group was significantly increased compared with the control group. The serum Cr and BUN levels in the Dox group were also higher than those in the control group (Figure 1(c)). The results in Figure 1(d) showed that the histopathological changes of the kidney in mice caused by Dox were found compared with the control group.

### 3.2. Dox Aggravates Oxidative Damage and Apoptosis *In Vitro* and *In Vivo*

**Figure 2:**
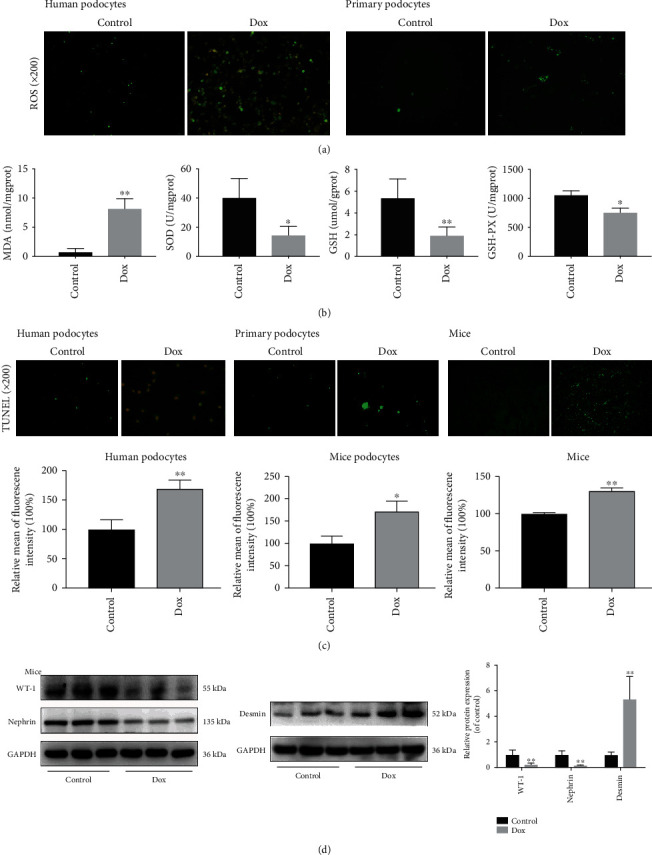
Dox aggravates oxidative damage and apoptosis *in vitro* and *in vivo*. (a) Intracellular ROS levels in human podocytes and mouse primary podocytes (*n* = 3). (b) The levels of MDA, SOD, GSH, and GSH-Px in kidney tissues of mice (*n* = 8). (c) Cell apoptosis based on TUNEL assay *in vitro* and *in vivo*. (d) The protein levels of WT-1, nephrin, and desmin in kidney tissues of mice by western blotting assay (*n* = 3). Values are listed as the mean ± SD. ^∗^*p* < 0.05 and ^∗∗^*p* < 0.01 compared with control groups.

The results in Figure 2(a) showed a significant increase in intracellular ROS level in human podocytes after treatment with Dox (32 *μ*M) for 24 h, and Dox at the concentration of 20 *μ*M for 12 h treatment obviously increased the ROS level in mouse primary podocytes. The data in Figure 2(b) showed that the MDA level was evidently increased compared with the control group, and the levels of SOD, GSH, and GSH-Px were obviously decreased in kidney tissues of the Dox group. In addition, the results in Figure 2(c) showed that Dox significantly increased the numbers of apoptotic cells *in vitro* and *in vivo*. As shown in Figure 2(d), compared with the control group, the expression levels WT-1 and nephrin were obviously decreased, and the expression level of desmin was evidently increased in the Dox group.

### 3.3. MsrB3 Is the Target Gene of miR-874-3p

**Figure 3:**
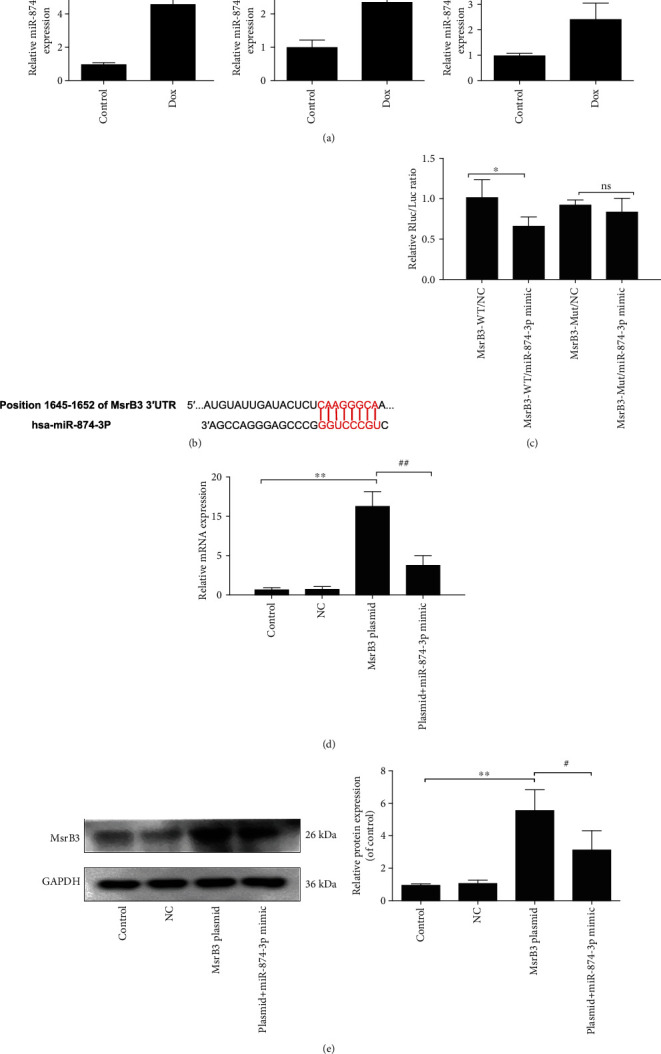
MsrB3 is the target gene of miR-874-3p. (a) miR-874-3p levels in human podocytes, mouse primary podocytes, and mice based on real-time PCR assay. (b) The diagram of miR-874-3p conservative seed binding sites on the 3′-UTRs of the target genes (MsrB3). (c) The results of the double luciferase reporter assay. (d, e) The expression levels of MsrB3 after cotransfection. All data are listed as the mean ± SD (*n* = 3). ^∗^*p* < 0.05 compared with control groups; ^∗^*p* < 0.05 compared with the NC group; ^#^*p* < 0.05 compared with the MsrB3 plasmid group; ns: no significance.

Dox significantly increased the expression level of miR-874-3pin cells and mice (Figure 3(a)). The binding site between MsrB3 3′-UTR mRNA and miR-874-3p was confirmed (Figure 3(b)). Double luciferase reporter assay results in Figure 3(c) certified that the relative luciferase expression of the miR-874-3p mimic+MsrB3-WT group was evidently decreased compared with the miR-874-3p mimic negative control (NC)+MsrB3-WT group. However, the effect was not observed with the mutated MsrB3 group, suggesting that MsrB3 has a specific binding site with miR-874-3p. The results of cotransfection (Figures 3(d) and 3(e)) indicated that the expression of MsrB3 both in protein and mRNA levels was obviously reduced in cotransfection miR-874-3p mimic and the MsrB3 overexpression plasmid group compared with single transfection of the MsrB3 overexpression plasmid group, indicating that miR-874-3p downregulated the expression level of MsrB3.

### 3.4. Dox Downregulates the Levels of MsrB3 *In Vitro* and *In Vivo*

**Figure 4:**
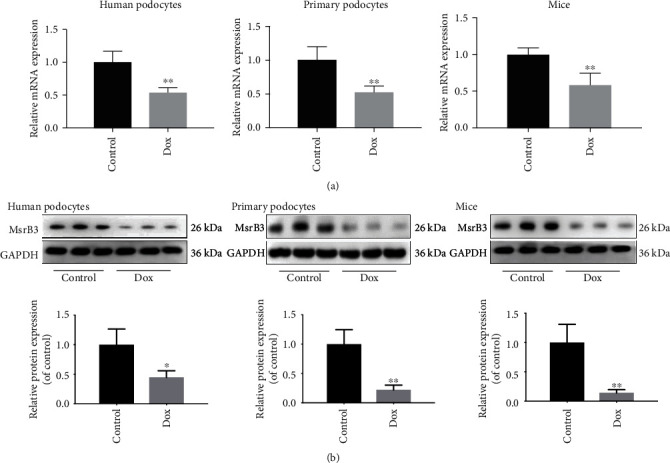
Dox downregulates the levels of MsrB3 *in vitro* and *in vivo*. (a) The mRNA levels of MsrB3 in human podocytes, mouse primary podocytes, and mice by real-time PCR assay. (b) The protein levels of MsrB3 *in vitro* and *in vivo* by western blotting assay. All data are expressed as the mean ± SD (*n* = 3). ^∗^*p* < 0.05 compared with control groups.

The data in Figure 4(a) illustrated that Dox significantly reduced the mRNA levels of MsrB3 *in vitro* and *in vivo* compared with control groups. Western blotting assay showed that the protein level of MsrB3 in the Dox group was lower than that in the control group (Figure 4(b)).

### 3.5. miR-874-3p Adjusts the MsrB3 Signal Pathway

**Figure 5:**
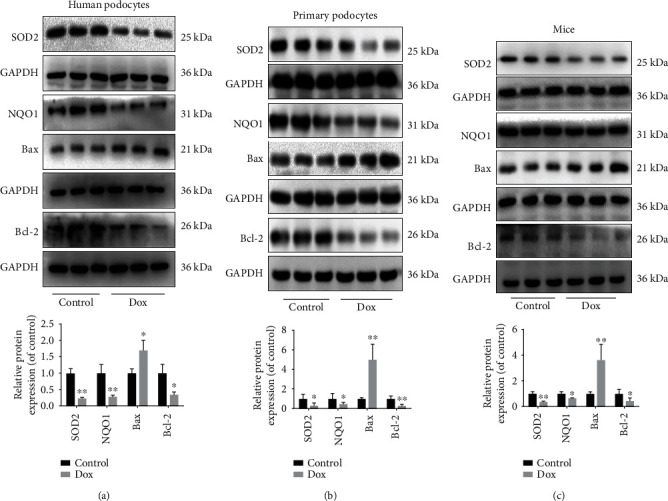
miR-874-3p adjusts the MsrB3 signal pathway. (a) The protein levels of SOD2, NQO1, Bax, and Bcl-2 in human podocytes. (b) The protein levels of SOD2, NQO1, Bax, and Bcl-2 in mouse primary podocytes. (c) The protein levels of SOD2, NQO1, Bax, and Bcl-2 in BALB/c mice. Values are listed as the mean ± SD (*n* = 3). ^∗^*p* < 0.05 compared with control groups.

The results in Figures 5(a)–5(c) illustrated that the expression levels of NQO1, SOD2, and Bcl-2 were obviously decreased, and Bax levels were evidently increased by Dox in human podocytes, mouse primary podocytes, and kidney tissues of mice compared with control groups.

### 3.6. Blockade of miR-874-3p Mitigates Dox-Caused Injury *In Vitro*

**Figure 6:**
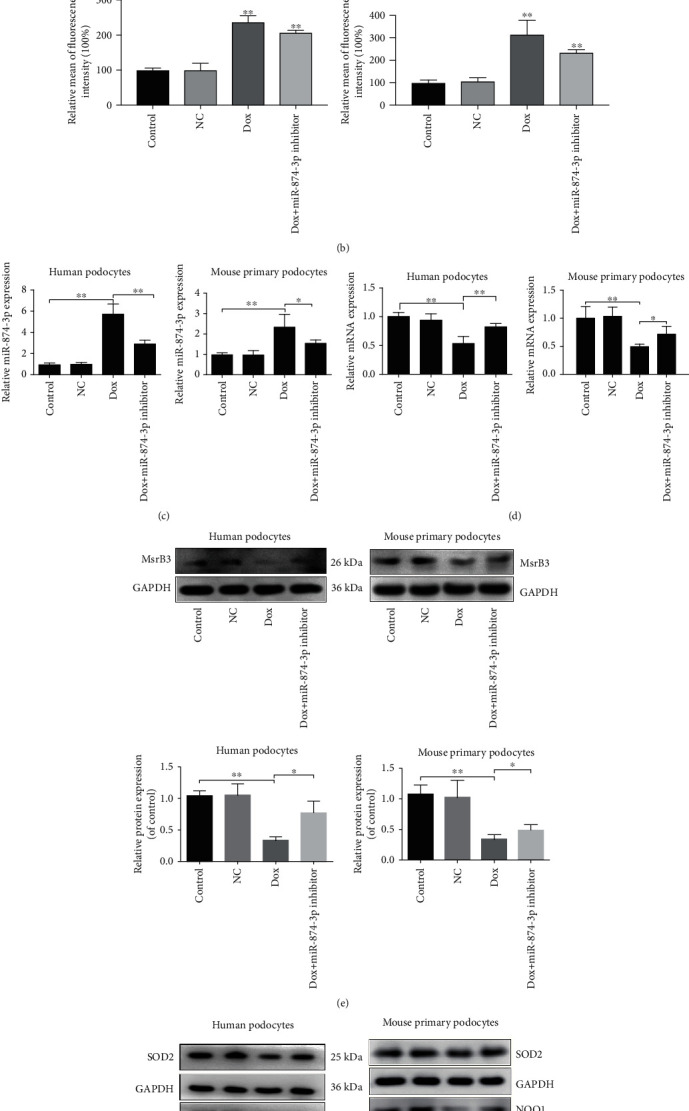
Blockade of miR-874-3p mitigates Dox-caused injury *in vitro*. (a) Intracellular ROS level in human podocytes and mouse primary podocytes. (b) Cell apoptosis in human podocytes and mouse primary podocytes. (c) The levels of miR-874-3p in human podocytes and mouse primary podocytes. (d) MsrB3 mRNA levels in human podocytes and mouse primary podocytes. (e) MsrB3 protein levels in human podocytes and mouse primary podocytes. (f) The protein levels of SOD2, NQO1, Bax, and Bcl-2 in human podocytes and mouse primary podocytes. Values are listed as the mean ± SD (*n* = 3). ^∗^*p* < 0.05 and ^∗∗^*p* < 0.01 compared with NC groups or compared with Dox groups.

As shown in Figure 6(a), ROS levels in human podocytes and mouse propodocytes were significantly decreased after transfection with miR-874-3p inhibitor compared with Dox groups. The results in Figure 6(b) illustrated that the apoptotic cells were reduced in Dox+miR-874-3p inhibitor groups compared with Dox groups. After transfection with miR-874-3p inhibitor, miR-874-3p levels were decreased in cells compared with Dox groups (Figure 6(c)). In addition, the mRNA and protein levels of MsrB3 were increased after transfection with miR-874-3p inhibitor compared with Dox groups (Figures 6(d) and 6(e)). The protein levels of Bax were decreased, and the expression levels of SOD2, NQO1, and Bcl-2 were increased in Dox+miR-874-3p inhibitor groups compared with Dox groups (Figure 6(f)).

### 3.7. Blockade of miR-874-3p Alleviates Dox-Induced Kidney Damage *In Vivo*

**Figure 7:**
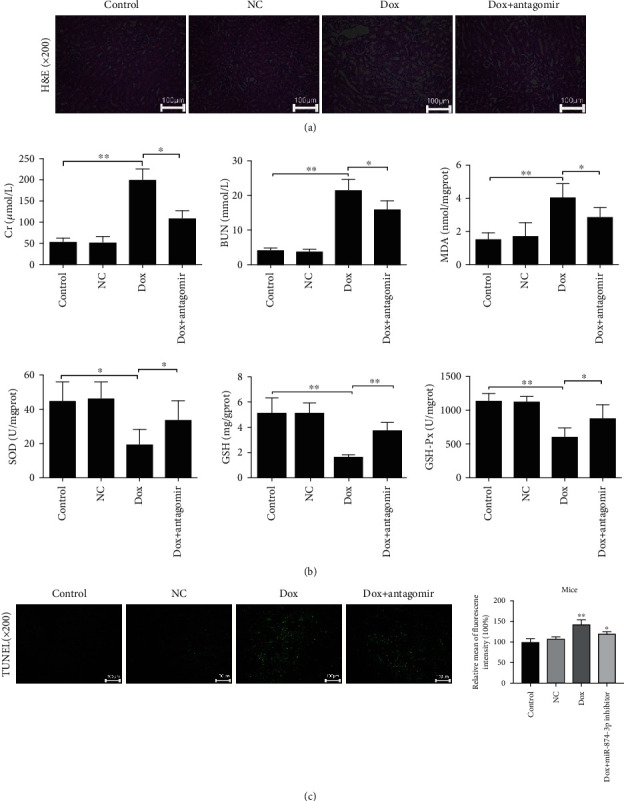
Blockade of miR-874-3p mitigates Dox-induced kidney injury *in vivo*. (a) The results of H&E staining of kidney tissues in Dox-treated mice after administered with miR-874-3p antagomir. (b) The levels of Cr, BUN, MDA, SOD, GSH, and GSH-Px in mice after treated with miR-874-3p antagomir. (c) Cell apoptosis in mice after treated with miR-874-3p antagomir. Values are listed as the mean ± SD (*n* = 5). ^∗^*p* < 0.05 and ^∗∗^*p* < 0.01 compared with the NC group or the Dox group.

As shown in Figure 7(a), administering with miR-874-3p antagomir in mice markedly mitigated histopathological injury induced by Dox. The data in Figure 7(b) showed that compared with the Dox group, Cr, BUN, and MDA levels were obviously decreased, and SOD, GSH, and GSH-Px levels were notably increased in the Dox+miR-874-3p antagomir group. The data in Figure 7(c) expounded that compared with the Dox group, cell apoptosis was reduced after treatment with miR-874-3p antagomir.

### 3.8. Blockade of miR-874-3p Alleviates Dox-Induced Renal Podocyte Injury *In Vivo*

**Figure 8:**
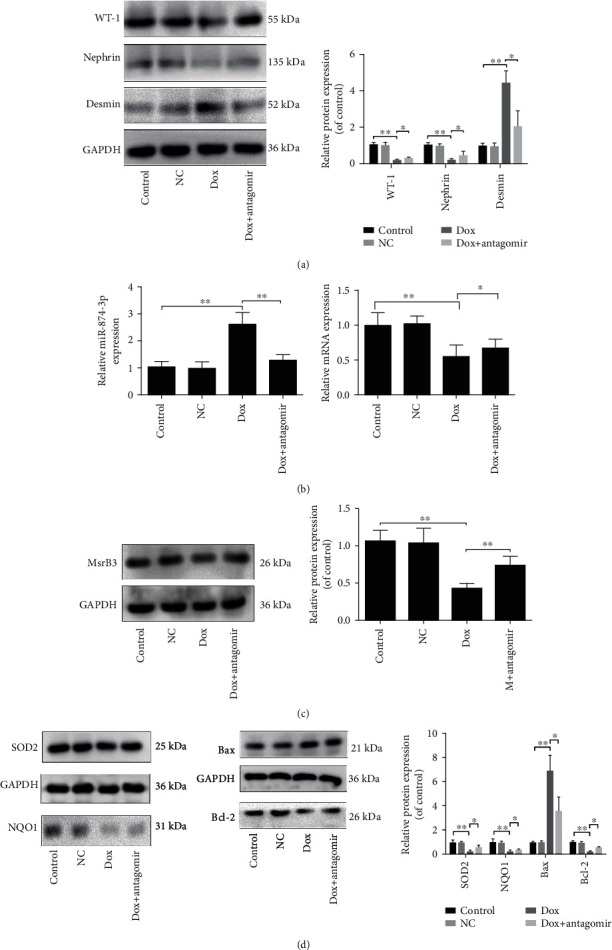
miR-874-3p mitigates Dox-induced renal podocyte injury via adjusting MsrB3 signal after antagomir test in mice. (a) The expression levels of WT-1, nephrin, and desmin after blockade of miR-874-3p by western blotting assay. (b) The expression levels of miR-874-3p and MsrB3 by real-time PCR assay in the kidney of mice after treatment with miR-874-3p antagomir. (c) The protein level of MsrB3 after treatment with miR-874-3p antagomir based on western blotting assay. (d) The protein levels of SOD2, NQO1, Bax, and Bcl-2 after administration with miR-874-3p antagomir *in vivo*. Values are listed as the mean ± SD (*n* = 3). ^∗^*p* < 0.05 compared with the Dox group.

The data in Figure 8(a) indicated that compared with the Dox group, in the Dox+miR-874-3p antagomir group, the protein levels of WT-1 and nephrin were obviously increased, and the desmin level was significantly decreased. Compared with the Dox group, the miR-874-3p level was obviously decreased and the mRNA level of MsrB3 was obviously increased in the Dox+miR-874-3p antagomir group (Figure 8(b)). The results in Figure 8(c) showed that the inhibition of miR-874-3p decreased the protein level of MsrB3. In addition, the results in Figure 8(d) showed that the expression levels of SOD2, NQO1, and Bcl-2 were significantly increased in the Dox+miR-874-3p antagomir group, and the Bax expression level was obviously reduced compared with the Dox group.

### 3.9. Blockade of MsrB3 Aggravates Dox-Caused Injury *In Vitro*

**Figure 9:**
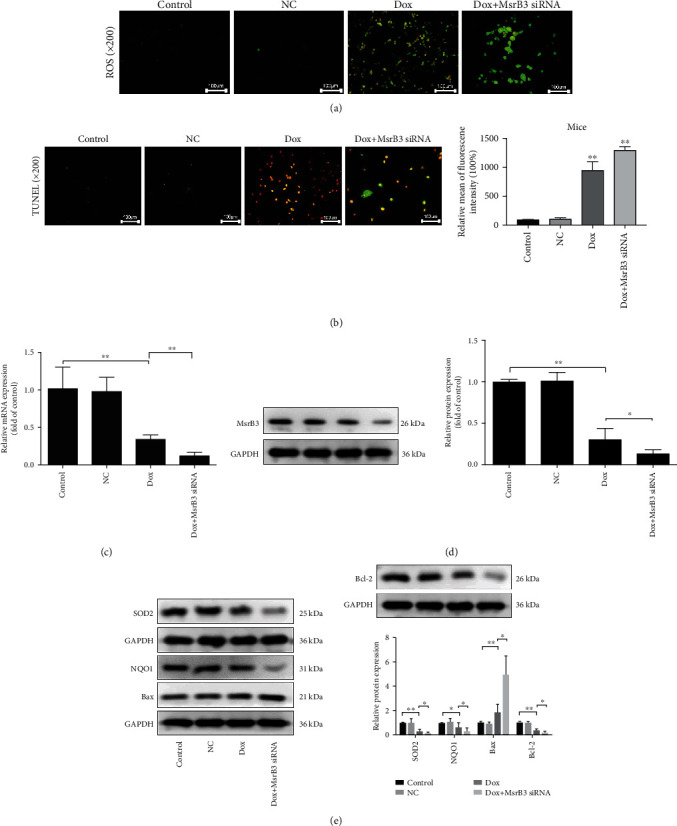
Blockade of MsrB3 aggravates Dox-caused injury *in vitro*. (a) Intracellular ROS level in podocytes after transfection with MsrB3 siRNA. (b) Cell apoptosis after transfection of MsrB3 siRNA. (c) The expression level of MsrB3 after transfection with MsrB3 siRNA in human podocytes based on real-time PCR assay. (d) The protein level of MsrB3 after transfection of MsrB3 siRNA by western blotting assay in human podocytes. (e) The protein levels of SOD2, NQO1, Bax, and Bcl-2 after transfection of MsrB3 siRNA in human podocytes. Values are listed as the mean ± SD (*n* = 3). ^∗^*p* < 0.05 compared with the Dox group or the NC group.

As shown in Figure 9(a), after transfection with MsrB3 siRNA, the level of ROS was increased in human podocytes, and the results in Figure 9(b) showed that a large number of apoptotic cells occurred in the Dox+MsrB3 siRNA group compared with the Dox group. The mRNA level of MsrB3 was significantly reduced (Figure 9(c)), and compared with the Dox group, the protein level of MsrB3 was obviously reduced in the Dox+MsrB3 siRNA group (Figure 9(d)). As shown in Figure 9(e), the Bax expression level was obviously increased in the Dox+MsrB3 siRNA group, and the expression levels of SOD2, NQO1, and Bcl-2 were evidently decreased compared with the Dox group.

## 4. Discussion

Cancer is the second leading cause of death in the world [36]. Anticancer therapy can destroy the physiological balance and affect the function of multiple organs [37]. As mentioned above, the clinical application of Dox is limited due to its multiple side effects, and the toxic effects of it on the renal structure can increase glomerular permeability and lead to proteinuria [38, 39]. In this study, we found that Dox significantly augmented the levels of Cr and BUN, downregulated the levels of WT-1 and nephrin, upregulated desmin expression, and aggravated the histopathological changes of the kidney in mice. In addition, Dox changed the cellular structure and decreased the viabilities of human podocytes and mouse primary podocytes. These results indicated that Dox induced renal podocyte injury *in vivo* and *in vitro*.

The injury and loss of podocyte have been confirmed as important markers in the pathogenesis of glomerular injury [40]. Some studies have shown that oxidative injury and cell apoptosis play key roles in Dox-induced renal podocyte injury. Some biological indicators including MDA, SOD, GSH, and GSH-Px show critical functions in oxidative stress. In this work, we found that Dox induced high intracellular ROS levels, high MDA level, low levels of SOD, GSH, and GSH-Px in mice, and obvious cell apoptosis. Therefore, we confirmed that oxidative stress and apoptosis may be critical factors of aggravating Dox-induced renal podocyte injury.

In the past few decades, lots of miRNAs have been testified as the important regulators on various biological processes, and some miRNAs have participated in Dox-induced renal podocyte injury. In addition, miR-205 and miR-217 and other miRNAs can be considered as the markers for clinical diagnosis and treatment of diseases [41, 42]. Circulating serum miR-205 can be considered as a biomarker for the diagnosis of the damage induced by aminoglycoside antibiotics in mice [41]. miR-217 can be used as one useful diagnostic biomarker associated with human podocyte cell apoptosis [42]. miR-874 contributes to inhibit tumor angiogenesis through targeting STAT3 in gastric cancer [43], which can also inhibit cell proliferation and induce apoptosis in human breast cancer by targeting cyclin-dependent kinase 9 (CDK9) [44]. In the current study, we observed that the expression levels of miR-874-3p were significantly increased in Dox groups both *in vitro* and *in vivo* compared with control groups. In addition, miR-874-3p directly targeted MsrB3 and downregulated the expression level of MsrB3. Thus, we considered that miR-874-3p may serve as a marker in clinical diagnosis and treatment of Dox-induced renal podocyte injury via targeting MsrB3.

MsrB3, a zinc-containing enzyme, plays important functions in some diseases via modulating oxidative stress and apoptosis [45]. Thus, we explored the role of MsrB3 in Dox-induced renal podocyte injury in this study, and the results indicated that the expression levels of MsrB3 were obviously reduced in Dox groups compared with control groups *in vitro* and *in vivo.* Hence, we considered that MsrB3 may be a marker in Dox-induced renal podocyte injury.

To further explore the mechanisms of miR-874-3p on Dox-induced renal podocyte injury through inhibiting MsrB3, the transfection tests of miR-874-3p inhibitor in cells and miR-874-3p antagomir in mice were carried out. The results showed that after blocking miR-874-3p, the expression levels of miR-874-3p were reduced, and the expression levels of MsrB3 were increased to suppress oxidative stress and cell apoptosis. In addition, the test of MsrB3 siRNA in human podocytes showed that oxidative stress and cell apoptosis were increased after blockade of MsrB3. Thus, we concluded that blockade of miR-874-3p mitigated Dox-induced oxidative stress and apoptosis against renal podocyte injury via inhibiting MsrB3.

## 5. Conclusion

We found that miR-874-3p aggravated Dox-induced renal podocyte injury by regulating oxidative injury and apoptosis via inhibiting MsrB3. In addition, this study may highlight the clinical relevance of miR-874-3p, and miR-874-3p/MsrB3 may be considered as a new target for the diagnosis and treatment of Dox-induced renal podocyte injury.

## Data Availability

The data used to support the findings of this study are included within the article.

## Supplementary Materials

Supplementary Materials The primer sequences of miRNAs, the primer sequences used for a real-time PCR assay, and the information of the antibodies used in the present work. In addition, one graphic abstract was also included in it.
