# Supplementary material for: MicroRNA-874-3p Aggravates Doxorubicin-Induced Renal Podocyte Injury via Targeting Methionine Sulfoxide Reductase B3
Source: Oxid Med Cell Longev. 2020 Aug 18;2020:9481841. doi: 10.1155/2020/9481841 (PMC7450315; doi:10.1155/2020/9481841)
Supplement: Supplementary Materials — The primer sequences of miRNAs, the primer sequences used for a real-time PCR assay, and the information of the antibodies used in the present work. In addition, one graphic abstract was also included in it. [file 9481841.f1.docx]

**Supplementary information**

**Supplementary Table S1** The primers sequences of miRNAs in the present work.

| miRNAs/ID | Primer sequence (5′-3′) |
| --- | --- |
| hsa-miR-874-3p-F/ MIMAT0004911 | CTGCCCTGGCCCGAGGGACCGA |
| mmu-miR-874-3p-F/ MIMAT0004853 | CTGCCCTGGCCCGAGGGACCGA |

**Supplementary Table S2** The primer sequences used for real-time PCR assay in the present work.

| Gene | Forward primer (5’→3’) | Reverse primer (5’→3’) |
| --- | --- | --- |
| Homo sapiens |  |  |
| Human-MsrB3 | AACTGAGGAAGCGGCTAACA | ACAAGGCAGCCGAATTTATG |
| Human-GAPDH | GCACCGTCAAGGCTGAGAAC | TGGTGAAGACGCCAGTGGA |
| Mus musculus |  |  |
| Mouse-MsrB3 | TCGGAGGCCATCGAGTTC | CAAGGATGCTGAGTTGATGCA |
| Mouse-GAPDH | TGTGTCCGTCGTGGATCTGA | TTGCTGTTGAAGTCGCAGGAG |
|  |  |  |

**Supplementary Table S3** The information of the antibodies used in the present work.

| Primary antibody | Source | Dilution | Company |
| --- | --- | --- | --- |
| MsrB3 | Mouse | 1: 200 | Santa Cruz Biotechnology, Dallas, Texas, USA |
| WT-1 | Rabbit | 1: 1000 | Proteintech Group, Chicago, USA |
| Nephrin | Rabbit | 1: 200 | Boster Biological Technology, California, USA |
| Desmin | Rabbit | 1: 1000 | Proteintech Group, Chicago, USA |
| SOD2 | Rabbit | 1: 1000 | Proteintech Group, Chicago, USA |
| NQO1 | Rabbit | 1: 1000 | Proteintech Group, Chicago, USA |
| BAX | Rabbit | 1: 1000 | Proteintech Group, Chicago, USA |
| Bcl-2 | Rabbit | 1: 1000 | Proteintech Group, Chicago, USA |
| GAPDH | Rabbit | 1: 1000 | Proteintech Group, Chicago, USA |

**
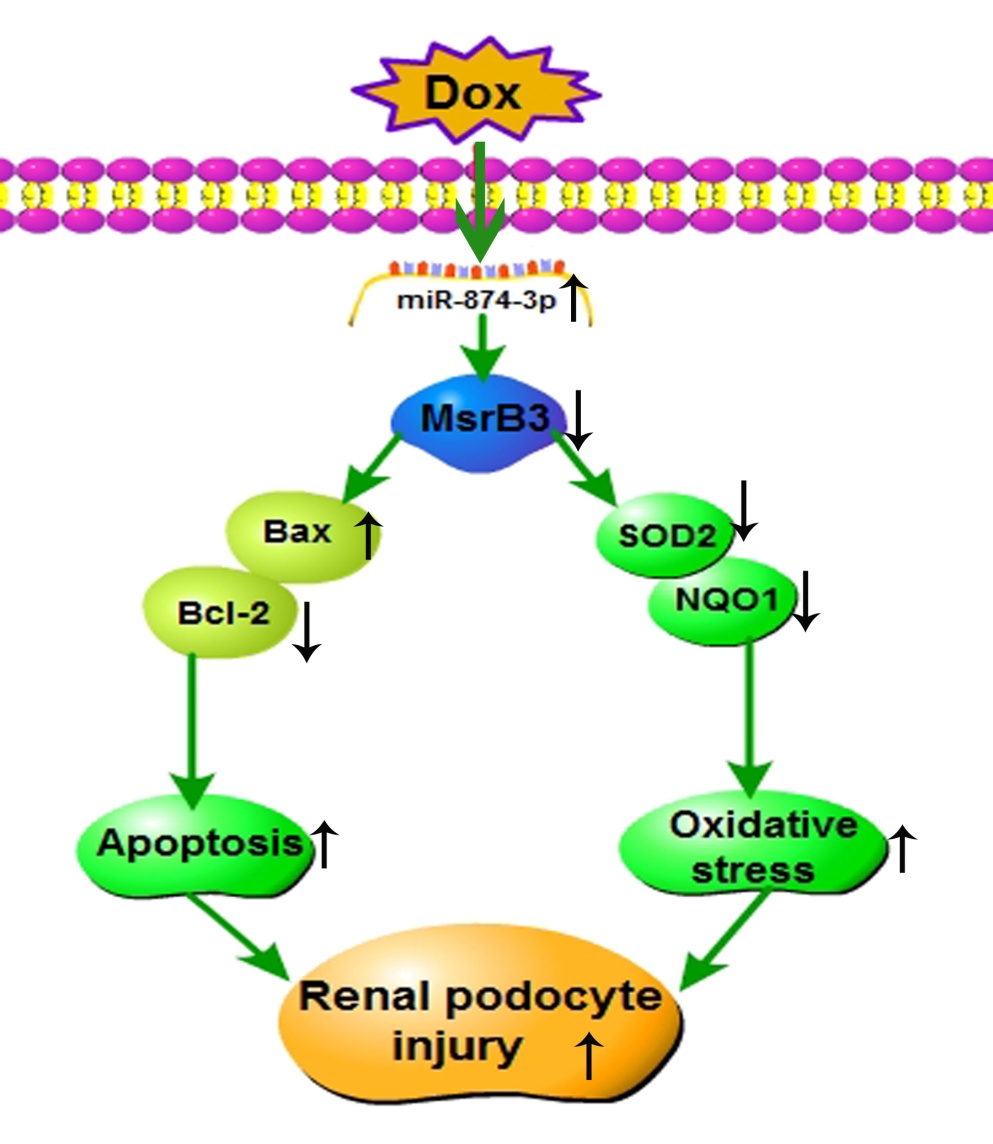
**

**Supplementary Figure S1** MiR-874-3p aggravated Dox-induced renal podocyte injury by promoting oxidative stress and apoptosis via targeting MsrB3.
